# Pediatric Headache Patients Are at High Risk of Vitamin D Insufficiency

**DOI:** 10.1177/08830738241284057

**Published:** 2024-10-09

**Authors:** Éloïse R. Deschênes, Jeffrey Do, Anne Tsampalieros, Richard J. Webster, Nicole Whitley, Leanne M. Ward, Daniela Pohl

**Affiliations:** 1 Undergraduate Medical Education, Faculty of Medicine, University of Ottawa, Ottawa, Ontario, Canada; 2 274065 Children's Hospital of Eastern Ontario Research Institute, Ottawa, Ontario, Canada; 3 Department of Pediatrics, 27338 Children's Hospital of Eastern Ontario, Ottawa, Ontario, Canada; 4 Division of Endocrinology and Metabolism, Department of Pediatrics, 27338 Children's Hospital of Eastern Ontario, University of Ottawa, Ottawa, Ontario, Canada; 5 Division of Neurology, Department of Pediatrics, 27338 Children's Hospital of Eastern Ontario, University of Ottawa, Ottawa, Ontario, Canada

**Keywords:** children, epilepsy, headache, migraine, other

## Abstract

**Background:**

Vitamin D deficiency has been associated with headaches in adults, but data for children with headaches are sparse.

**Objective:**

To describe vitamin D levels in children with headaches.

**Methods:**

We retrospectively analyzed serum 25(OH)D concentrations in children aged 2-17 years with headaches compared to children with epilepsy at the Children's Hospital of Eastern Ontario between October 1, 2014, and August 19, 2021. Serum 25(OH)D <50 nmol/L was classified as insufficient.

**Results:**

Vitamin D concentrations of 353 children (117 with headaches; 236 with epilepsy) were analyzed. The median age in years was 10 (interquartile range [IQR] 5, 14); 50.4% of subjects were female. The median serum 25(OH)D was 56 nmol/L (IQR 41, 69) in children with headaches and 70 nmol/L (IQR 50, 95) in children with epilepsy. Vitamin D insufficiency was present in 42% of children with headaches and 25% of children with epilepsy (*P *= .002). In a multivariable linear regression model adjusting for age, sex and seasonality, children with headaches had serum 25(OH)D concentrations that were on average 9 nmol/L (95% CI-16.76, −0.96) lower compared to children with epilepsy (*P *= .029).

**Conclusion:**

The prevalence of vitamin D insufficiency is higher in children with headaches compared to children with epilepsy. Prospective studies are needed to assess if vitamin D supplementation may have a therapeutic effect on pediatric headaches.

Headaches are a frequent complaint in children, with a worldwide pediatric prevalence of 54%.^1^ The exact pathophysiology of headaches remains unclear but is likely multifactorial with a genetic predisposition and environmental (including nutritional) factors as possible triggers. Headache-preventative pharmaceutical treatments for children often lack evidence.^2^ Headache prophylaxis using nutraceuticals has gained popularity, including magnesium and vitamin B_2_, with some studies demonstrating migraine preventative effects in the pediatric age group.^3^

There is growing interest in the effect of vitamin D on various health conditions, and there has been a recent increase in vitamin D insufficiency world-wide, particularly in countries with seasonal low sunlight index. Vitamin D deficiency has been associated with a variety of pain syndromes, including arthritis, muscle pain and chronic diffuse pain.^4^ Vitamin D supplementation, particularly in people who are deficient, has been reported to relieve pain.^5^

In adults, a lower vitamin D status has been associated with headaches,^6–14^ and vitamin D supplementation was found to be beneficial in reducing headache frequency.^15–17^ There is growing evidence that low vitamin D levels are also associated with headaches in children.^18–22^ Recent publications suggest that vitamin D supplementation may be beneficial in children with headaches.^19,23–26^ Supplementation with 2000 IU daily of vitamin D for 2 months followed by 600 to 1000 IU for 6 months resulted in a decrease in migraine frequency, duration, severity, and migraine-related disability in Turkish children.^19^ In a separate prospective trial, vitamin D supplementation in addition to amitriptyline was more effective than amitriptyline alone in reducing the frequency of migraine in children.^25^ Limitations of this study were its brief observation period, which did not cover all seasons, the absence of an analysis of the participants’ dietary habits, and the lack of a healthy control group.^25^ Overall, there currently is still insufficient evidence supporting an association between low vitamin D levels and headaches, and vitamin D supplementation has not yet been established as a nutraceutical treatment approach.

The biological mechanisms of vitamin D effects on pain relief are under investigation. Vitamin D receptors have been found in various parts of the brain.^27–29^ Vitamin D supplementation has been linked to changes in neurotransmitters including cholinergic transmission,^30^ noradrenaline,^31^ glutamine,^31^ dopamine,^31^ and serotonin.^31^ The antioxidant stress-reducing abilities of vitamin D may also have a neuroprotective effect.^32^

In our study, we aimed to describe vitamin D levels in Canadian children with headaches and determine their risk of vitamin D insufficiency. As we did not have access to a healthy control group, we assessed vitamin D levels in children with epilepsy, another common chronic neurologic disorder, as a comparator.

## Materials and Methods

### Participants

We conducted a retrospective chart review of children with headaches or epilepsy treated at the Children's Hospital of Eastern Ontario, in Ottawa, Canada between October 1, 2014, and August 19, 2021. We used October 1, 2014, as the cut-off because this was the date of the implementation of our electronic medical record system. We included children between 2 and 17 years of age. The children had either a diagnosis of headache or epilepsy. Headache diagnosis was based on the International Headache Society criteria.^33^ All types of headaches were included. The epilepsy diagnosis was based on the ILAE official report.^34^ Children who had serum 25-hydroxyvitamin-D (25(OH)D) testing performed no more than 3 months prior to, or any time after receiving a diagnosis of epilepsy or headaches were included. Children with more than 1 test were included only once in the analysis, with the test result closest to the time of diagnosis. Children with a concomitant headache and epilepsy diagnosis were excluded.

**Figure 1.**
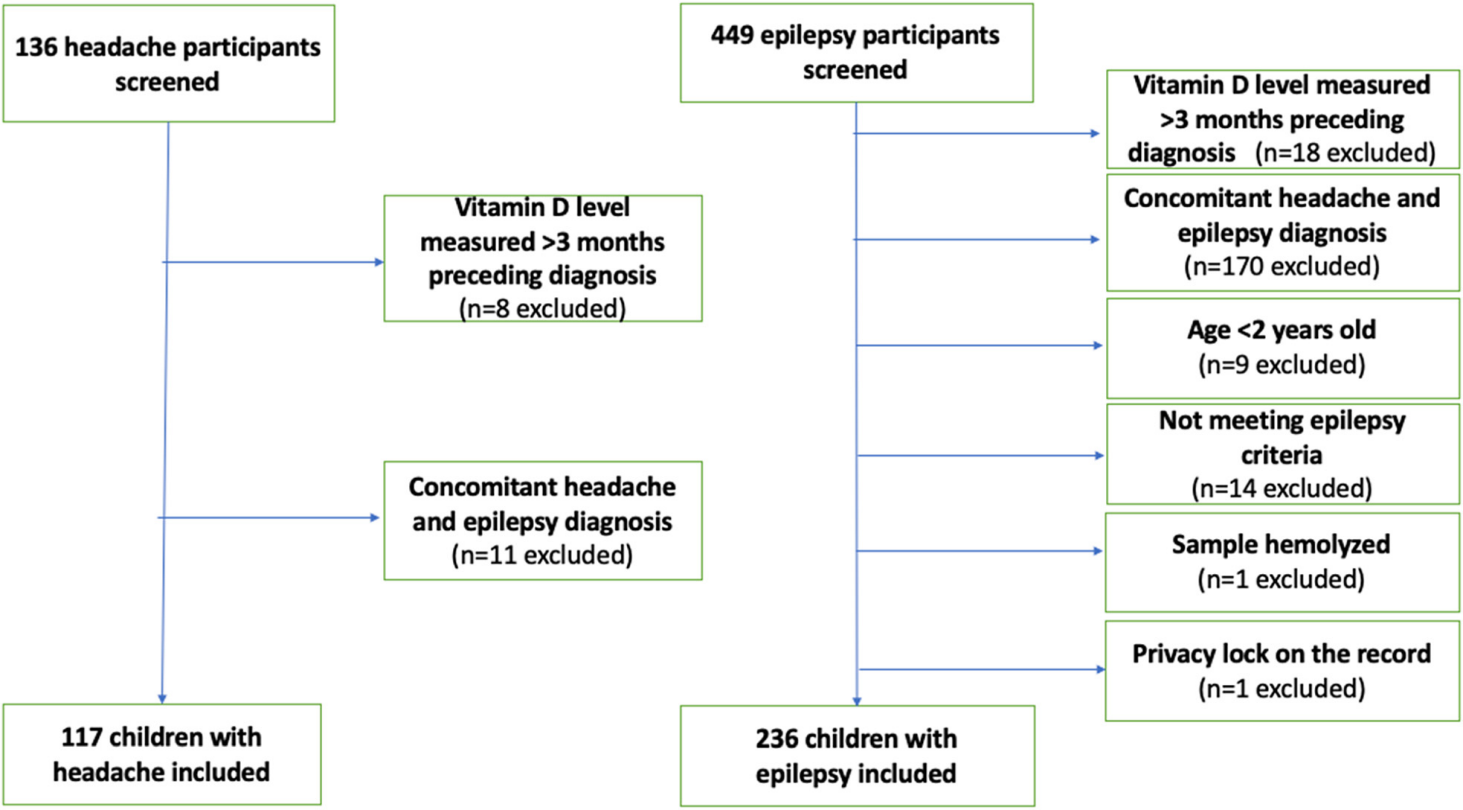
Flow diagram of screened and included participants.

A list of 585 children was provided by the Children's Hospital of Eastern Ontario data warehouse team, of which 353 met inclusion criteria (117 with headaches and 236 with epilepsy). Two children were excluded from the epilepsy group: 1 whose serum sample was hemolyzed and 1 whose medical record was locked for privacy reasons. A total of 353 participants (117 with headaches and 236 with epilepsy) were included in the study. See Figure 1 for complete details of reasons for exclusion.

### Methods

#### Outcome

All 25(OH)D concentrations were measured using immunoassays. Between October 1, 2014, and July 24, 2017, all samples were tested via the IDS-iSYS analyzer. From July 24, 2017, to November 27, 2019, samples were analyzed using the Beckman Dxl analyzer. Since November 27, 2019, the Roche Elecsys immunoassay has been used at The Ottawa Hospital (TOH). Liquid chromatography–tandem mass spectrometry (LC-MS/MS) methods are considered the gold standard for assessing 25(OH)D concentrations.^35^ Research has examined the strength of the relationship between LC-MS/MS and the techniques applied to our samples. The *r* values for the IDS-iSYS, Roche Elecsys immunoassay, and the Beckman Dxl analyzer were respectively 0.93, 0.96-0.99, and 0.94, rendering all applied methods comparable.^36–38^

The primary outcome of interest was serum 25(OH)D concentration. For descriptive purposes, vitamin D thresholds used for this study were based on the guidelines for bone health: serum concentrations ≥50 nmol/L were classified as sufficient,^39^ and levels <50 nmol/L were considered insufficient.

Covariates of interest included age at the time of bloodwork in years, sex, and seasonality. Seasonality at time of bloodwork was treated as a dichotomous variable based on high ultraviolet (UV) light index (April 1 until October 31) or low UV index (November 1 until March 31) in Canada.

### Statistical Methods

All analyses were performed using the R statistical programming language (version 4.0.5).^40^ We tested for an association between serum 25(OH)D concentration (primary outcome) and the diagnosis (headaches and epilepsy), while adjusting for age, sex, and seasonality. Age, a continuous variable, was tested for linearity, and as it was nonlinear, a restricted cubic spline was fitted.^41^ A Wilson score with 95% confidence intervals (CIs) was calculated for the proportion of participants with vitamin D insufficiency. A χ^2^ test of proportions was used to test for differences. The primary analysis was a multivariable regression model.

## Results

### Demographics

We included 353 children, and the majority (67%, n = 236) had a diagnosis of epilepsy. Children with headaches represented 33% (n = 117) of the cohort. Overall, there were more females than males included in the study (Table 1).

**Table 1.** Demographics of Children With Headaches or Epilepsy.

	Overall (n = 353)	Headaches (n = 117)	Epilepsy (n = 236)
Median age, years (IQR*)	10 (5, 14)	14 (10, 15)	8 (4, 12)
Male, n (%)	(49.6)	42 (35.9)	133 (56.4)
Female, n (%)	(50.4)	75 (64.1)	103 (43.6)

Abbreviation: IQR, interquartile range.

### Serum 25(OH)D Concentration

**Figure 2.**
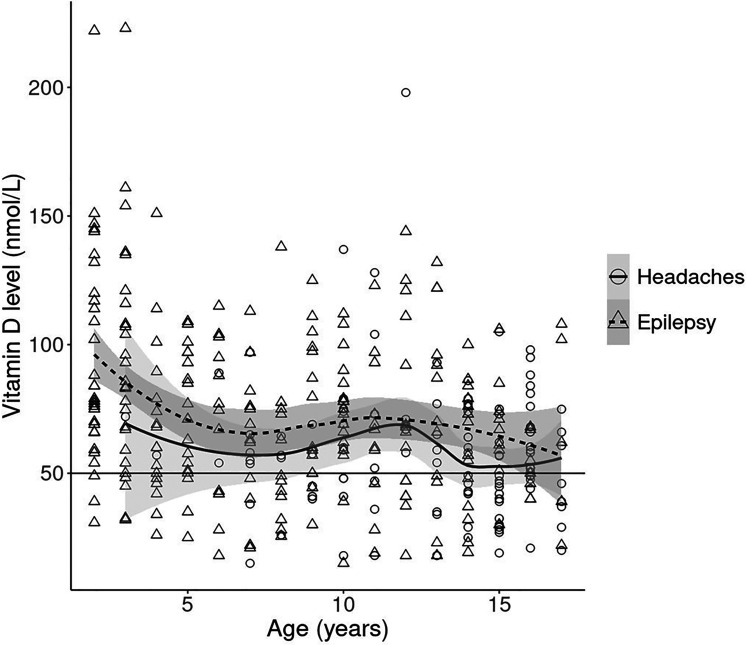
Relationship between age in years and serum 25(OH)D (nmol/L) level by diagnosis. The curves represent age-related means of serum vitamin D levels for headache patients (solid) and epilepsy patients (dotted). Curves were obtained by using a locally estimated scatterplot smoothing (LOESS). The gray shading around the curves represents 95% confidence intervals. The circles and triangles are individual levels from patients with headaches and epilepsy. The horizontal black line shows the vitamin D insufficiency cutoff (50 nmol/L).

The median serum 25(OH)D concentration was lower in children with headaches when compared to children with epilepsy (Table 2). The relationship between age and serum 25(OH)D was similar in both children with headaches and those with epilepsy (Figure 2).

**Table 2.** Serum 25(OH)D concentration (nmol/L) in children with headaches or epilepsy.

Median (IQR*), 25(OH)D nmol/L			
	Overall (n = 353)	Headaches (n = 117)	Epilepsy (n = 236)
Whole group	65.0 (47.0, 85.0)	56.0 (41.0, 69.0)	70.0 (49.8, 95.2)
Male	67.0 (48.0, 84.5)	57.5 (42.2, 70.5)	69.0 (50.0, 89.5)
Female	62.0 (46.1, 84.8)	54.1 (41.0, 69.0)	71.0 (49.5, 97.0)

Abbreviation: IQR, interquartile range.

The covariate age was found to have a nonlinear relationship with serum 25(OH)D levels, and a 4-knot restricted cubic spline was included in the model. There was evidence of an association between diagnosis and vitamin D level in a multivariable model after adjusting for age, season, and sex. Children with headaches had serum 25(OH)D concentrations 8.9 nmol/L (95% CI 16.76, 0.96) lower than our comparison group of children with epilepsy (Table 3).

**Table 3.** Multivariable Modeling for Serum 25(OH)D Levels.^a^

Covariate	Adjusted *β* (95% CI)	*P* value
Diagnosis: Headaches (reference = epilepsy)	−8.9 (−16.76, −0.96)	.029
Sex: Male (reference = female)	−0.52 (−7.19, 6.14)	.878
Season^b^: High UV index (reference = low UV index)	2.31 (−4.17, 8.79)	.485

^a^ Age is also adjusted for in the multivariable model and results are presented in supplementary material.

^b^ High UV index refers to serum collected between April 1 until October 31, and low UV index between November 1 until March 31.

### Seasonality and Serum 25(OH)D Concentration

**Figure 3.**
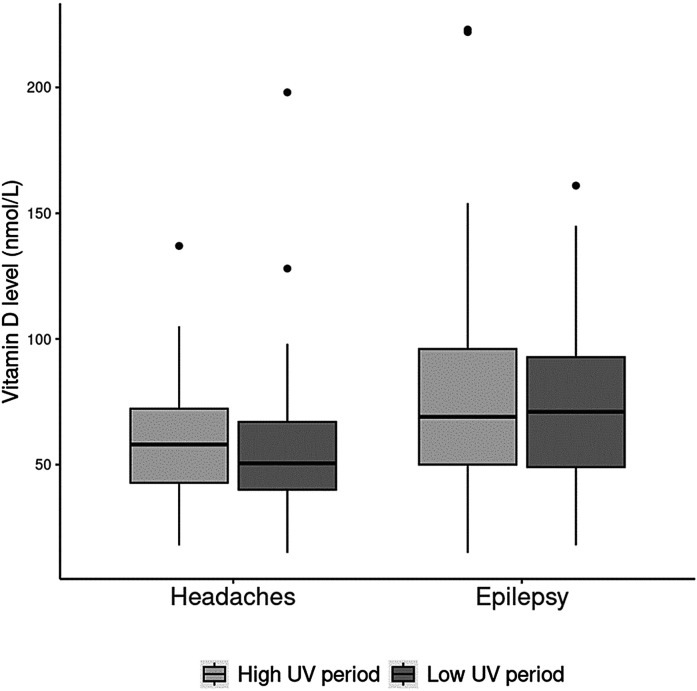
Illustrates the average 25(OH)D (nmol/L) level in relation to seasonal high (April 1 to October 31) versus low (November 1 until March 31st) ultraviolet (UV) light index in children with headaches or epilepsy. For this box plot the median, IQR, and 95% CI are represented by (i) the horizontal line within the box, (ii) the top and bottom of the box, and (iii) the upper and lower limit of the vertical bar.

The distribution of serum 25(OH)D levels was similar by season in children with headaches and epilepsy (Figure 3). The median (IQR) vitamin D levels for patients with headaches during high UV periods was 58.0 (42.8, 72.2) nmol/L and for low UV periods 50.5 (40.0, 67.0) nmol/L. The median (IQR) vitamin D level for patients with epilepsy and high UV period was 69.0 (50.0, 96.0) nmol/L and for low UV period 71.0 (49.0, 92.8) nmol/L.

### Prevalence of Vitamin D Insufficiency

**Figure 4.**
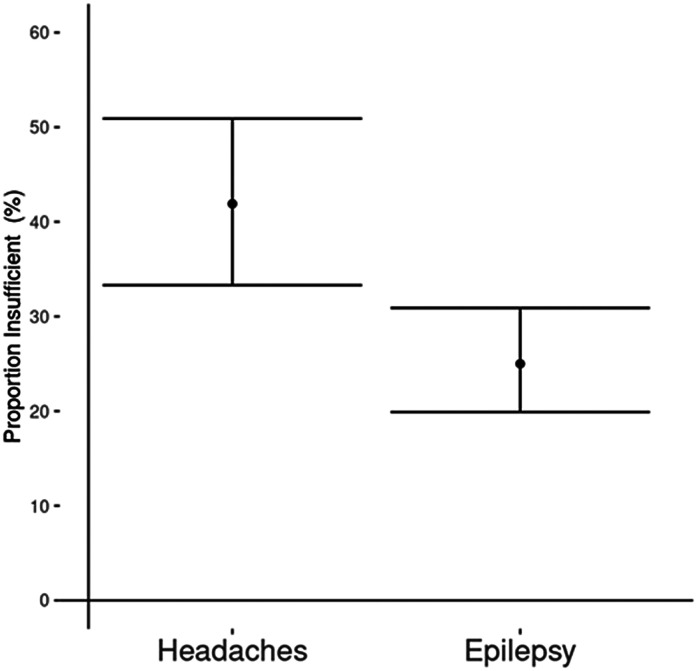
Proportion of children with vitamin D insufficiency in the headache and the epilepsy group (percentage with Wilson score 95% CI).

Vitamin D levels were dichotomized into 2 categories, insufficient (<50 nmol/L) versus sufficient (≥50 nmol/L). There was a higher percentage of children with headaches and vitamin D insufficiency (41.9%, 95% CI 33.3, 50.9) compared with children with epilepsy (25.0%, 95% CI 19.9, 30.9) (*P *= .002) (Figure 4).

## Discussion

Our study demonstrates that vitamin D levels are lower in children with headaches than in children with epilepsy. This difference persisted after we adjusted for age, sex, and seasonality.

For this retrospective study, we did not have access to a healthy pediatric cohort. We decided to assess the vitamin D status in children with epilepsy as a comparator because most of those patients will have vitamin D level assessments as standard of care at our center. Individuals with epilepsy are known to have lower vitamin D levels than healthy individuals.^42^ Vitamin D insufficiency was found in 25% of our epilepsy participants. This is consistent with published data from the United States and Thailand, which reported 25(OH)D levels <50 nmol/L in 25% and 23% of children with epilepsy, respectively.^43,44^ Surprisingly, when compared to children with epilepsy, our children with headaches had vitamin D concentrations that were on average 9 nmol/L lower. To our knowledge, this is the first time these 2 populations are being compared.

Because we were not able to directly compare our data to vitamin D levels in healthy children at our center, we reviewed published data on vitamin D levels for Canadian children on a population basis. The Canadian Health Measure Survey (CHMS) has been reporting vitamin D levels since 2007 based on random volunteer samples. In 2007-2011, 23% of children had vitamin D serum levels <50 nmol/L.^45^ In our study, levels below 50 nmol/L were present in 42% of headache patients, demonstrating a higher frequency of vitamin D insufficiency in our headache group than in the general Canadian pediatric population. However, this comparison must be interpreted with caution, because data were collected during different time intervals, at diverse latitudes, and vitamin D levels were measured with different methods.

Interestingly, the 42% incidence of vitamin D insufficiency in our cohort of pediatric headache patients is in keeping with a retrospective study from Turkey, a country with higher UV radiation, where 46% of children with migraine were vitamin D insufficient.^19^ In another retrospective analysis of children with headaches from Turkey, 69% had serum 25(OH)D levels <50 nmol/L.^20^ However, a case-control study conducted in Iran including 105 headache participants and 110 controls aged 15-65 years found no significant difference in the vitamin D status between the 2 groups, with 80% of the headache participants and 82% of the controls showing vitamin D insufficiency.^46^ Given the extremely high overall prevalence of vitamin D insufficiency in both control and patient groups, a review group hypothesized that external factors like the fact that most Iranian women wear covering clothing and receive little UV exposure may have obscured the potential difference between the groups.^47^

Vitamin D status varies with sun exposure.^48^ During winter, the skin produces very little vitamin D, especially at latitudes higher than 35°.^49^ Our patient catchment area (around Ottawa, Ontario) is located at latitudes of 44° to 46°, resulting in limited UV exposure during the winter. Surprisingly, in our study, vitamin D levels measured during higher UV index months were not significantly higher when compared to levels measured during low UV index months. We hypothesize that the observed lack of seasonal variation in pediatric vitamin D levels is a consequence of the fact that Canadian children and adolescents spend less time outside than they did in the past.^50^ Furthermore, the COVID-19 pandemic is included in our study's time frame. According to a pediatric study conducted in the United States, outdoor activity participation overall decreased by 22% during the pandemic.^51^ Of note, a Canadian study reported that during the pandemic, children's recreational screen time increased by about 11 hours per week.^52^ These factors could help explain why seasonality did not influence the vitamin D levels in our study: Canadian children spend most of their time indoors, across all seasons.

There was a nonlinear association between age and vitamin D level in our cohort, with a decreasing trend. A study from Southeastern China demonstrated similar findings, with adolescents being at higher risk of vitamin D insufficiency as compared to younger school-aged children.^53^ A Turkish study also found a negative correlation between age and 25(OH)D concentrations.^54^ These findings could be interpreted in the context of age-related lifestyle changes, with teenagers often having more screen time and being more sedentary,^55^ and therefore spending less time outdoors. According to Canadian guidelines, children older than 5 years should limit their screen time to 2 hours per day.^56^ However, a 2017 Canadian study reported that only 8% of adolescents met the screen time recommendations.^56^ Another potentially contributing factor is that older Canadian children consume less vitamin D–fortified milk than younger children.^57^

**Figure 5.**
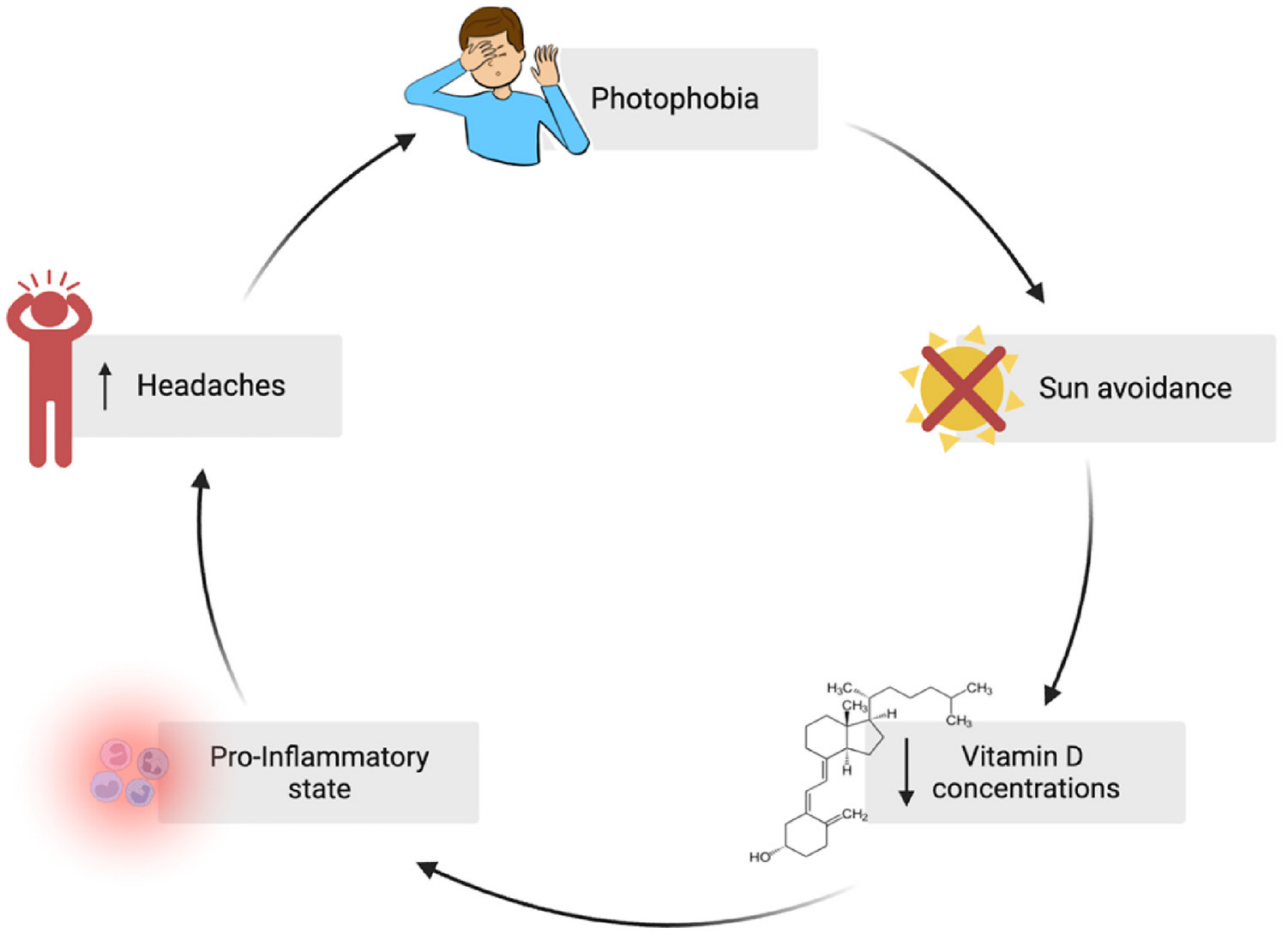
Hypothesis of a vicious cycle with photophobia as a pivotal factor for vitamin D insufficiency in patients with headaches. Created with BioRender.com.

Our study did not analyze potential reasons for vitamin D insufficiency in pediatric headache patients. However, we hypothesize that headaches may interfere with the frequency and duration of outdoor activities. Headaches are frequently associated with photophobia, triggering an avoidance of sun exposure. This may lead to a consecutive lack of UV-induced endogenous vitamin D synthesis. A vicious cycle may ensue: lower vitamin D concentrations are associated with increased pro-inflammatory states in children.^58^ Pro-inflammatory processes are discussed as one of several pathophysiological trigger mechanisms in headaches,^17,59^ and an increased headache frequency may then lead to further sun avoidance and even lower endogenous vitamin D synthesis (Figure 5). Supporting this hypothesis, a recent study in migraine patients aged 15-55 years demonstrated that the incidence of photophobia was higher in individuals with lower vitamin D concentrations compared to those with normal levels.^8^ Although our pathophysiologic model (Figure 5) is still hypothetical and covers only 1 aspect of headache pathogenesis, it may suggest a potential benefit of vitamin D supplementation in children with headaches. However, we cannot exclude that low vitamin D levels in children with headaches are just a consequence of the preference of children with headaches to stay indoors, without a negative effect of vitamin D insufficiency on headaches. Future studies are necessary to determine the impact of vitamin D supplementation on headaches.

Our study is limited because of its retrospective design, and therefore not all variables affecting serum vitamin D levels were captured (eg, ethnicity, skin pigmentation, recent travel, diet, vitamin D supplements, medication usage, or sun exposure). Given the nature of this study, we were not able to determine the type of headache diagnosis although we have observed at our tertiary care center that the majority of referred children are diagnosed with migraine. Most studies cited do discuss migraine specifically. Another limitation of our study is that not all children who visited Children's Hospital of Eastern Ontario for headaches or epilepsy underwent bloodwork to assess vitamin D levels, thereby creating a potential selection bias.

## Conclusion

Our study found that a higher proportion of children with headaches (42%) than children with epilepsy (25%) had an insufficient 25(OH)D status. Children with headache had on average a serum 25(OH)D level 9 nmol/L lower than children with epilepsy. Future studies should aim to prospectively assess the vitamin D status in children with headaches and assess the potential effects of vitamin D supplementation on pediatric headache frequency and intensity.

## Supplemental Material

sj-docx-1-jcn-10.1177_08830738241284057 - Supplemental material for Pediatric Headache Patients Are at High Risk of Vitamin D Insufficiency Supplemental material, sj-docx-1-jcn-10.1177_08830738241284057 for Pediatric Headache Patients Are at High Risk of Vitamin D Insufficiency by Éloïse R. Deschênes, MD, Jeffrey Do, MD, Anne Tsampalieros, MD, PhD, Richard J. Webster, PhD, Nicole Whitley, BSc, Leanne M. Ward, MD, and Daniela Pohl, MD, PhD in Journal of Child Neurology
