# Supplementary material for: Pediatric Headache Patients Are at High Risk of Vitamin D Insufficiency
Source: J Child Neurol. 2024 Oct 9;40(2):91–8. doi: 10.1177/08830738241284057 (PMC11783970; doi:10.1177/08830738241284057)
Supplement: sj-docx-1-jcn-10.1177_08830738241284057 - Supplemental material for Pediatric Headache Patients Are at High Risk of Vitamin D Insufficiency [file sj-docx-1-jcn-10.1177_08830738241284057.docx]

**Supplementary material**

**Justification for RCS**

**Restricted cubic spline was used to model the nonlinear age effect where knots for age: 2.0, 7.0, 12.0, 16.0.**

 Age* (4-knot restricted cubic spline) years

  1st coefficient:  -8.01 (-11.72, -4.30)

  2nd coefficient: 17.55 (7.23, 27.86)

  3rd coefficient: -49.16 (-80.04, -18.28)

<0.001
